# New Appliances & Things Medical

**Published:** 1909-07-03

**Authors:** 


### New Appliances & Things Medical.

[We shall be glad to receive at our Office, 28 & 29 Southampton Street, Strand, London, W.C., from the manufacturers, specimens of all new preparations and appliances.]

### FLAVOURED SANATOGEN.

(The Sanatogen Co., 12 Chenies Street, London, W.C.)

Sanatogen is now so well known to the medical profession as a therapeutic and dietetic agent that further description of it is unnecessary beyond the reminder that it contains assimilable organic phosphorus. Certain patients, however, object to the flavour of the ordinary Sanatogen. It is well known that the taste of a substance and the immediate psychical sensations to which it gives rise have always some influence, and in neurotic patients an important influence, upon its digestibility. In order to prevent the flavour of Sanatogen diminishing its range of utility its manufacturers have now put upon the market a "Flavoured Sanatogen." The result in our opinion is satisfactory. The flavour is distinct, but not too pronounced, and the new article should be an acquisition to those medical practitioners whose patients included some who disliked the natural flavour of the old Sanatogen and therefore shrank from using it.

